# Caspase-3 and Caspase-6 cleave STAT1 in leukemic cells

**DOI:** 10.18632/oncotarget.1911

**Published:** 2014-04-18

**Authors:** Verena Licht, Katrin Noack, Bernhard Schlott, Martin Förster, Yvonne Schlenker, Andreas Licht, Oliver H. Krämer, Thorsten Heinzel

**Affiliations:** ^1^ Leibniz Institute for Age Research – Fritz Lipmann Institute, Beutenbergstrasse 11, 07745 Jena, Germany; ^2^ Friedrich-Schiller-Universität Jena, Centre for Molecular Biomedicine (CMB), Institute for Biochemistry and Biophysics, Hans-Knöll-Str. 2, 07745 Jena, Germany; ^3^ Integrated Research and Treatment Center, Center for Sepsis Control and Care (CSCC), Jena University Hospital, , Erlanger Allee 101, 07747 Jena, Germany; ^4^ Internal Medicine I, Pulmonary Medicine and Allergy/Immunology, University Clinics Jena, Erlanger Allee 101, 07747 Jena, Germany; ^5^ Jena Bioscience GmbH, Loebstedter Strasse 80, 07749 Jena, Germany; ^6^ Institut für Toxikologie, Universitätsmedizin Mainz, Obere Zahlbacher Str. 67, 55131 Mainz, Germany

**Keywords:** Apoptosis, Caspase-3, Caspase-6, HDACi, Leukemia, STAT1

## Abstract

Signal Transducer and Activator of Transcription-1 (STAT1) is phosphorylated upon interferon (IFN) stimulation, which can restrict cell proliferation and survival. Nevertheless, in some cancers STAT1 can act in an anti-apoptotic manner. Moreover, certain malignancies are characterized by the overexpression and constitutive activation of STAT1. Here, we demonstrate that the treatment of transformed hematopoietic cells with epigenetic drugs belonging to the class of histone deacetylase inhibitors (HDACi) leads to the cleavage of STAT1 at multiple sites by caspase-3 and caspase-6. This process does not occur in solid tumor cells, normal hematopoietic cells, and leukemic cells that underwent granulocytic or monocytic differentiation. STAT1 cleavage was studied under cell free conditions with purified STAT1 and a set of candidate caspases as well as with mass spectrometry. These assays indicate that unmodified STAT1 is cleaved at multiple sites by caspase-3 and caspase-6. Our study shows that STAT1 is targeted by caspases in malignant undifferentiated hematopoietic cells. This observation may provide an explanation for the selective toxicity of HDACi against rapidly proliferating leukemic cells.

## INTRODUCTION

STAT proteins control the development and homeostasis of metazoans [1]. STATs are latent transcription factors that reside in the cytoplasm until they are activated by extracellular ligands. These are e.g., cytokines, growth factors, and hormones. Binding of these ligands allows the multimerization of their receptors. Subsequently, Janus tyrosine kinases (JAKs) are recruited to the cytoplasmic domains of these receptors and become activated by phosphorylation. JAKs directly phosphorylate STATs leading to a conformational switch of the homo- and heterodimers. These translocate into the nucleus, where they alter gene expression patterns [1]. Moreover, Stark and coworkers showed that STAT1 and STAT3 can modulate gene expression independent of their phosphorylation status [2].

Seven STAT proteins have been identified in mammals: STAT1, -2, -3, -4, -5a, -5b, and -6 [3, 4]. STAT1 has a primary role in IFN-mediated cell signaling and is important for processes like cell proliferation, survival, and immunological control [5]. While STAT1 can act as a tumor suppressor, an oncogenic potential of STAT1 has been shown in certain cellular contexts [6]. Furthermore, STAT1 overexpression is found in many leukemic cells and in some solid cancers [7]. Moreover, overexpressed, unphosphorylated STAT1 regulates a different subset of genes than phosphorylated STAT1, and these genes are often related to tumorigenesis [8, 9]. Hematopoietic cells are key cells of the immune system, which makes it important to analyze the stability of STAT1 in HDACi-treated normal and transformed blood cells.

Apoptosis (programmed cell death) is a critical barrier to tumorigenesis [10]. During apoptosis, activated caspases are the main executor enzymes that selectively cleave proteins. They are a multifunctional, highly conserved enzyme family. Caspases harbor a cysteine residue in their active center and cleave selectively after aspartate residues [11, 12]. Caspases are synthesized as immature zymogens requiring specific proteolysis for activation [13]. They can be generally subdivided into two classes. Initiator caspases are activated upon recruitment to multi-protein complexes like death receptors (caspase-8 and caspase-10) or the apoptosome (caspase-9). These caspases activate executioner or effector caspases [14], which cleave various proteins to allow cellular demise [12, 15]. In addition, several non-apoptotic functions of caspases have been identified in the last years, which indicates that these enzymes play important roles in cellular differentiation and proliferation [16]. Recent studies show that the activation of caspases also leads to selective protein degradation independent of apoptosis in hematopoietic cells [17].

The signaling of STAT1 is well studied but only a few analyses concerning the degradation and recycling of STAT1 were done. Kim and Maniatis reported that STAT1 becomes ubiquitinylated after IFNγ stimulation [18]. Other studies also show that STAT1 can be ubiquitinylated and degraded by the proteasome to restrict IFN-dependent JAK-STAT signaling [19, 20]. Remarkably, some pathogens like the SV5 or Mumps virus inactivate STAT1 via the induction of its proteasomal degradation [21, 22]. On the other hand, there is a positive feed-back loop on STAT1 expression, with IFNs activating STAT1 and the subsequent binding of STAT1 to its own promoter [23-25].

Little is known about the cellular turnover of STAT1 in uninduced cells. So far, STAT1 was identified as a caspase-3 target in HeLa cells [26]. Treatment with staurosporine, as classical apoptosis inducer, resulted in a C-terminally truncated STAT1 protein (STAT1γ) unable to induce IFN signaling. A complete *in vitro* degradation of STAT1 by caspase-3 was shown in cell-free extracts prepared from Jurkat cells which were treated with 50 mg/ml cytochrome c and 1mM dATP [27]. The above named studies indicate that STAT1 can be a substrate of caspase-3. However, it has not been formally addressed if caspases other than caspase-3 cleave STAT1 in cells.

HDACs are epigenetic modulators that catalyze the deacetylation of lysine residues [28]. Inhibition of these enzymes with HDACi modulates several functions of the immune system. Of note, STAT1 signaling is not exclusively regulated by phosphorylation, but equally by acetylation [29]. Several studies show that HDACi modulate the acetylation of STAT1 and its transcriptional activity [30, 31]. The treatment of cells with HDACi alters protein degradation, signaling, gene expression, and apoptosis [32-34]. Accordingly, HDACi also are potent apoptosis inducers in certain cell types [28]. While HDACi block IFN-dependent STAT1 signaling, STAT1 expression is increased in melanoma and other solid cancer-derived cells when they are incubated with HDACi [25, 31, 35, 36].

We addressed whether HDACi affect the stability of STAT1 in leukemic cells and in normal blood cells. Our data show that treatment with HDACi induces apoptosis and allows the cleavage and degradation of STAT1. Furthermore, we reveal that STAT1 is a direct target of caspase-3 and caspase-6 in undifferentiated leukemic cells. Hormonally and chemically induced differentiation protects transformed cells from apoptosis involving the caspase-dependent processing of STAT1. The same holds true for normal blood cells. These results provide further understanding to the differential response of normal and leukemic cells to HDACi.

## RESULTS

### The expression of STAT1 in NB4 cells is reduced upon exposure to the HDACi butyrate

**Figure 1:**
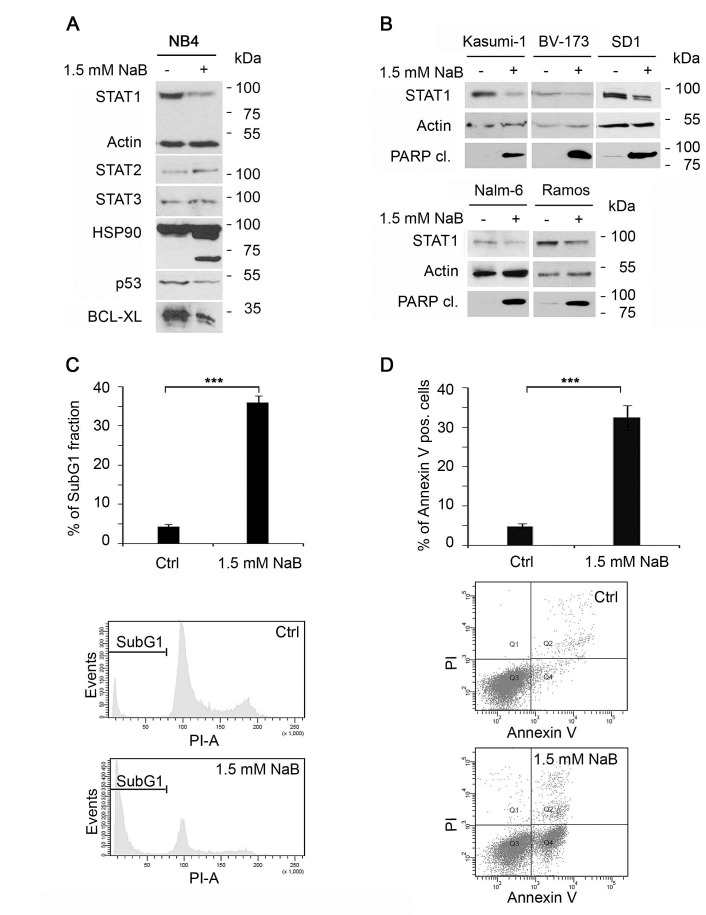
Butyrate alters STAT1 levels and expression of its target genes in NB4 cells A) Sodium butyrate (NaB) downregulates the expression of STAT1, but not STAT2 and STAT3 during apoptosis. NB4 cells were stimulated with NaB (1.5 mM) for 24 hours. Levels of endogenous STAT1, Actin (loading control), STAT2, STAT3, HSP90, p53 and BCL-XL were monitored by immunoblot. HSP90 cleavage indicates caspase activation leading to apoptosis. B) NaB leads to STAT1 degradation in leukemia cells (Kasumi-1, BV-173, SD1, Nalm-6 and Ramos). Leukemia cell lines were stimulated with 1.5 mM butyrate for 24 hours. STAT1, tubulin and PARP1 cleavage were monitored by immunoblot.C+D) NaB leads to apoptosis within 24 hours. NB4 cells were stimulated with 1.5 mM NaB and either stained with propidium-iodine for cell cycle profiling or with AnnexinV/ propidium-iodine. C) 54.8 % of cells were found in the SubG1 fraction after NaB treatment for 24 hours, respectively. D) In comparison 57.5 % + 5.3 % of the cells stained with AnnexinV/PI were seen. (means +/- SE; ***p < 0.001; n=3).

To determine whether HDACi affect the expression and activity of STAT1 in leukemic cells, we treated NB4 acute promyelocytic leukemia (APL) cells with butyrate, a naturally occurring HDACi. We found that butyrate treatment significantly reduces STAT1 levels in NB4 cells (Figure 1A). Since all STAT proteins share a high degree of homology [4], we also examined STAT2 and STAT3 protein levels in butyrate-treated cells. Whereas STAT2 was even slightly induced, STAT3 seemed to be unaffected by HDACi (Figure 1A and Supplemental Figure 1.1). Thus, of the STATs tested, specifically STAT1 becomes reduced after exposure of NB4 cells to butyrate.

The HDACi-induced attenuation of STAT1 is unexpected, as HDACi treatment results in an induction of STAT1 mRNA and protein levels in solid tumor derived cells [25, 31, 35, 36]. Therefore, we compared the effect of butyrate on various lymphoid and myeloid leukemia cells and on solid tumor-derived cells. Whereas butyrate reduces STAT1 in leukemia cells (Figure 1B), most solid tumor-derived cells show an induction of STAT1 after treatment (Supplemental Figure 1.2).

Since HDACi can activate caspases and the apoptotic program [37, 38], we tested whether butyrate has a pro-apoptotic effect on NB4 cells. A loss of full-length caspase-3 indicates its activation, i.e. the cleavage from the precursor into the active enzyme. An additional control for caspase activation is the detection of its cleaved substrates [11, 12]. These can for example be the DNA repair enzyme PARP1 and heat shock protein 90 (HSP90), both being direct targets of caspases in apoptotic NB4 cells [39]. Cleavage products of HSP90 and PARP1 can be detected in butyrate-treated NB4 cells (Supplemental Figure 1.1). In addition, the protein levels of mutant p53 become reduced (Figure 1A) and). Accordingly, the levels of the anti-apoptotic protein BCL-XL and BCL-2, which are positively regulated by mutant p53 [40, 41], are reduced in NB4 cells exposed to butyrate (Figure 1A and Supplemental Figure 1.1).

The HDACi butyrate induces significant amounts of apoptosis in NB4 cell cultures (Figure 1C). Apoptotic DNA fragmentation below a DNA content of 2N (SubG1 fraction of fixed cells) is detected in 32.4%+/-3.1% of the cells treated with 1.5 mM butyrate for 24 hours. Furthermore, 35.6%+/-1.7% of Annexin V-positive, i.e. apoptotic cells can be measured by AnnexinV/PI staining of unfixed cells (Figure 1D).

Data provided in Figure 1 show that STAT1 expression can be reduced by butyrate in leukemia cells. This loss of STAT1 appears to be linked induction of apoptosis by HDACi.

### Butyrate-induced downregulation of STAT1 correlates with apoptosis

**Figure 2:**
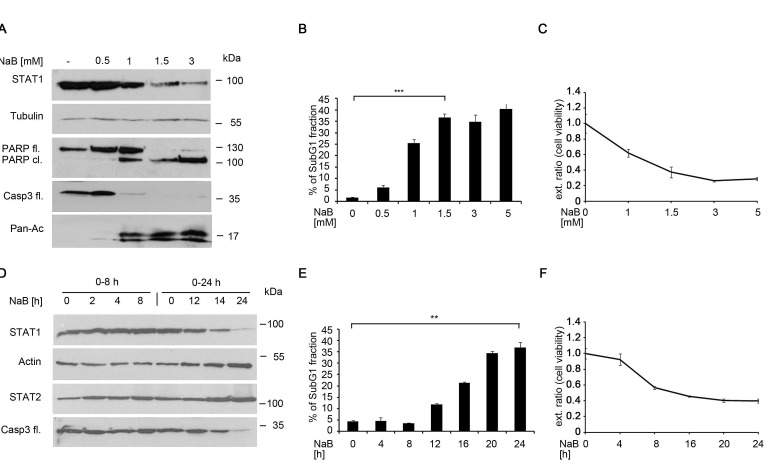
The HDACi butyrate induces time- and dose dependent STAT1 and apoptosis A) NB4 cells were left untreated or stimulated with NaB for 24 hours. Amounts of STAT1, caspase-3 full-length (fl.), PARP full- length and PARP cleaved form and the loading control Tubulin were determined by Western blot analysis. B) NaB induces apoptosis dose-dependently NB4 cells were stimulated with 0-5 mM NaB, stained with propidium-iodine and analyzed as described in Figure 1C (means +/- SE; n=3; ***p <0.001).C) Cells were stimulated with 0-5 mM NaB for 24 hours. Cell viability was determined by MTT assay. Cells which were left untreated were used as negative controls, respectively. D) NB4 cells were stimulated with 1.5 mM NaB for different time points over 24 hours. The protein levels of STAT1, STAT2, caspase-3 full length, and Actin as loading control were determined by Western blot analyses E) Butyrate induces apoptosis time-dependently. NB4 cells were stimulated with 1.5 mM butyrate for 24 hours, stained with propidium-iodine and analyzed as described in Figure 1C(means +/- SE; n=3; **p <0.01).F) NB4 cells were stimulated with 1.5 mM NaB at the indicated intervals. After 24 hours, MTT assay was performed as described in Figure 2C

Next, we investigated at which concentrations butyrate reduces STAT1 protein expression and whether this process can be correlated with the induction of apoptosis. Western blot experiments demonstrated that STAT1 degradation starting at a concentration of 1 mM butyrate (Figure 2A). Conversion of full-length caspase-3 and PARP1 cleavage can also be seen at this and higher concentrations of butyrate. In order to test for HDACi efficacy, we analyzed hyperacetylation of histones. Both, the induction of cell death as well as histone hyperacetylation correlate with the loss of STAT1 in NB4 cells exposed to butyrate (Figure 2A).

FACS analyses revealed that butyrate leads to an increase of the apoptotic cell fraction in a dose-dependent manner (Figure 2B) and MTT assays show that butyrate reduces cell viability of NB4 cells (Figure 2C). Increasing concentrations of butyrate has little additional effect; from 1.5 mM on a plateau in cell death, PARP1 cleavage, histone hyperacetylation, and loss of STAT1 expression is reached (Figure 2A-C).

We then determined the kinetics of STAT1 degradation in butyrate-treated cells with time course experiments for up to 24 hours. While the treatment with 1.5 mM butyrate for up to eight hours has no significant effect on STAT1 expression, treatment lasting from 12 hours on reduces STAT1 expression. Together with STAT1 degradation, caspase-3 cleavage indicating its activation can be observed. STAT2 again remained stable in the presence of butyrate (Figure 2D).

In NB4 cells STAT1 becomes clearly reduced after 12 hours of butyrate exposure. We determined whether this process is associated with apoptosis. FACS data showed that, from a 12 hour treatment period on, butyrate starts to cause cell death (Figure 2E). Additionally, we performed an MTT assay to analyze the butyrate effect on NB4 cell viability at various time points and doses. Cells stopped proliferation and underwent apoptosis and this correlated with the degradation of STAT1 (Figure 2C and 2F).

These findings demonstrate that STAT1 levels are reduced time- and dose-dependently by butyrate treatment and this effect correlates with the induction of apoptosis. This effect begins after 12 hours of treatment, indicating that the butyrate-induced STAT1 loss is most likely an indirect effect.

### Caspases cleave STAT1 in apoptotic cells

**Figure 3:**
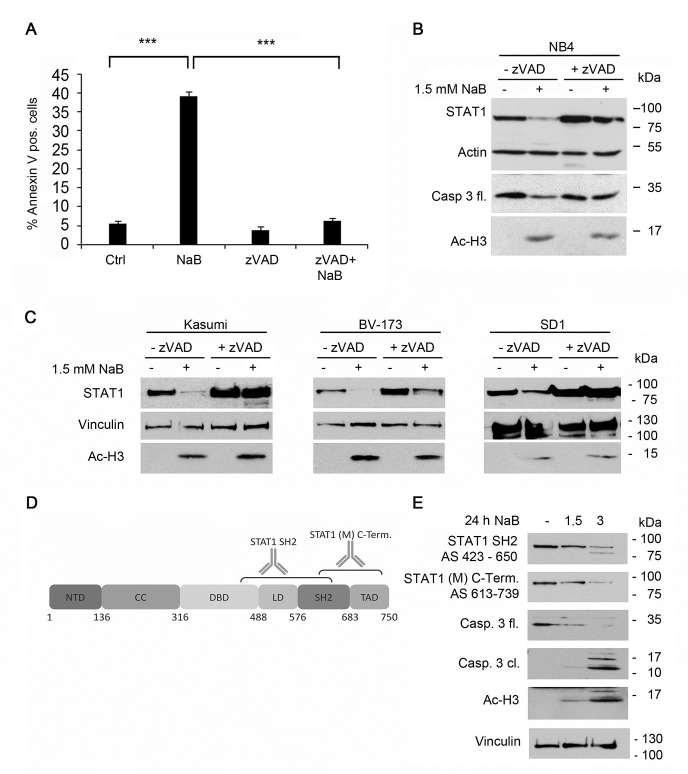
Decrease of STAT1 can be blocked by caspase inhibitors but not by protease- or proteasomal inhibitors A) Butyrate induced apoptosis can be prevented by Z-VAD-FMK. NB4 cells were preincubated with 20 μM Z-VAD-FMK for 1 hour and then stimulated with 1.5 mM NaB for 24 hours. They were stained and analyzed as described in Figure 1C. B) Z-VAD-FMK prevents degradation of STAT1. NB4 cells were stimulated with 1.5 mM NaB in the absence or presence of the pan-caspase inhibitor z-VAD-FMK as described in Figure 3A. The protein levels of STAT1, caspase-3 full length, acetylated histone H3 and β-actin as loading control were determined by Western Blot analyses. C) The hematopoietic cell lines Kasumi-1, SD1 and BV173 were stimulated with 3 mM NaB for 24 hours in absence or presence of a pre-stimulation with Z-VAD-FMK (50 μM Z-VAD-FMK was used for Kasumi; 25μM Z-VAD-FMK was used for SD1 and BV173). The protein levels of STAT1, acetylated histone H3 and vinculin as loading control were determined by Western Blot analyses. D) Schematic representation of STAT1 protein structure highlighting its functional domains and the epitopes of the STAT1 antibodies used in Figure 3E.E) NB4 cells were stimulated with 1.5 or 3 mM NaB for 24 hours. The protein levels of STAT1, caspase-3 full length (fl.) and cleaved (cl.), acetylated histone H3 and tubulin as loading control were determined by Western Blot analyses. Two different STAT1 antibodies which recognize different epitopes of STAT1 were used to detect the STAT1 cleavage product.

Our data suggest that butyrate promotes a caspase dependent processing of STAT1 in leukemic cells. In order to ascertain these findings, we blocked these enzymes with the pan-caspase inhibitor Z-VAD-FMK [12]. We pre-treated NB4 cells with Z-VAD-FMK and then added butyrate. FACS analyses of NB4 cells showed that Z-VAD-FMK significantly inhibits the butyrate-induced chromatin fragmentation (Figure 3A) as well as the cleavage of caspase-3 giving rise to its active form (Figure 3B). Of note, Western blot analyses revealed that Z-VAD-FMK blocks the degradation of STAT1 evoked by butyrate (Figure 3B). Detection of hyperacetylated histone H3 verified that the efficacy of butyrate is unaffected by Z-VAD-FMK.

Curiously, incubation of untreated NB4 cells with Z-VAD-FMK resulted in a slight increase in STAT1 protein levels. Hence, also the basal turnover of STAT1 is regulated by caspases in NB4 cells (Figure 3B).

The same effects were seen in the leukemia cell lines Kasumi-1, BV-173, and SD1. These findings illustrate that the caspase-dependent processing of STAT1 in response to butyrate treatment seems to be a general mechanism (Figure 3C).

The cleavage of proteins by caspases often leads to the appearance of protein fragments [12, 15]. We used several antibodies to detect such protein fragments of STAT1. Immunoblot data shown in Figures 1 and 2 were collected with a monoclonal anti-STAT1 antibody directed against the STAT1 C-terminus (Figure 3D). Since a C-terminal cleavage of STAT1 has been described [42], we also probed immunoblots with an antibody directed against more N-terminal residues (Figure 3D). This antibody (termed STAT1 SH2) revealed a fragment of STAT1 in addition to full-length STAT1 in NB4 cells exposed to butyrate (Figure 3E).

We did some additional tests to clarify whether other proteases contribute to the degradation of STAT1 in NB4 cells treated with butyrate. Various protease inhibitors could not prevent STAT1 degradation (Supplemental Figure S2.1). Since HDACi also induce proteasome-dependent protein degradation [33, 43], we tested whether the proteasome inhibitor lactacystin protects STAT1 from butyrate. This inhibitor could though not prevent STAT1 reduction (Supplemental Figure S2.2). The E3 ubiquitin-protein ligase RLIM is degraded by the proteasome after HDACi exposure [39]. The rescue of the butyrate-induced loss of RLIM by lactacystin served as control and verified the potency of the lactacystin batch (Supplemental Figure S2.2).

To test whether STAT1 degradation through treatment with butyrate requires *de novo* protein synthesis, experiments with the protein-synthesis inhibitor cycloheximide (CHX) in combination with butyrate were performed. After 24 hours, STAT1 protein levels were determined. Western blot analyses showed that treatment with butyrate as well as with CHX leads to reduced STAT1 levels after 24 hours (Supplemental Figure S2.3). These findings agree with STAT1 having a half-life between 12 and 24 hours [44, 45] and with the auto-regulatory control of STAT1 expression at the level of the *STAT1* promoter [23, 24]. The combination of cycloheximide and butyrate leads to a further reduction. Therefore, protein biosynthesis of an additional factor may not be required for the degradation of STAT1 in response to butyrate (Supplemental Figure S2.4). This is plausible as caspases are already present as precursors until they are activated.

Based on these data, we propose that STAT1 is a target of caspases during the HDACi-induced apoptosis.

### Caspase-3 and Caspase-6 cleave STAT1

**Figure 4:**
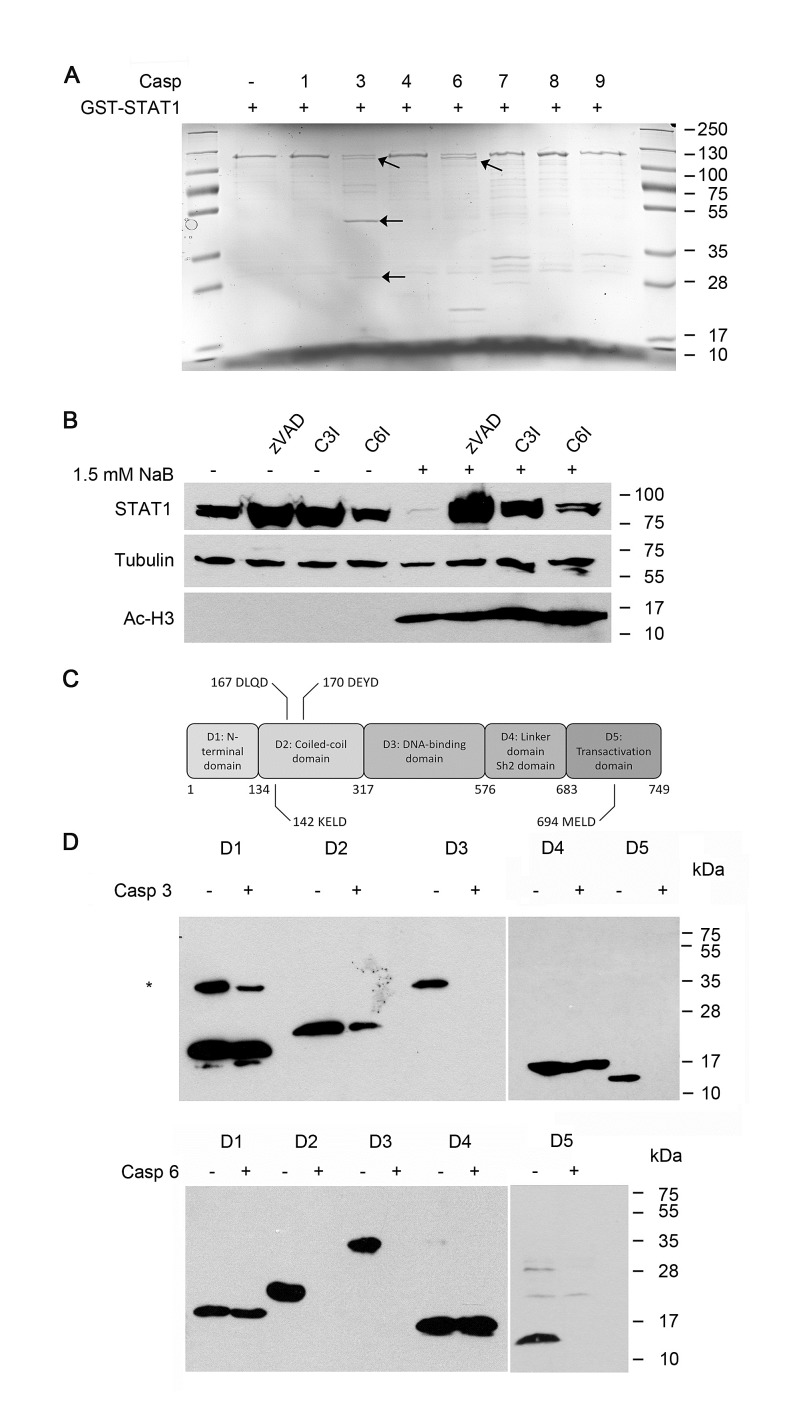
Cleavage of STAT1 by caspases.A) Caspase-3 and caspase-6, but not caspase 1, 4, 7, 8 and 9 cleave STAT1 Heterologous GST-STAT1 was purified and incubated with 1 unit of the indicated caspases for 3 hours at 37° C. Extracts were analyzed by SDS-PAGE and stained with Coomassie solution. Bands marked with arrows indicate cleavage products of STAT1. Bands in lane 7, 8 and 9 correspond to the molecular weight of the indicated caspases.B) STAT1 loss can be prevented by the usage of pan-caspase inhibitor as well as specific inhibitors for caspase-3 and caspase-6. NB4 cells were stimulated with 1.5 mM NaB in the absence or presence of the pan-caspase inhibitor z-VAD-FMK (20 μM), the caspase-3 inhibitor z-DEVD-FMK (20 μM; C3I) or the caspase-6 inhibitor z-VEID-FMK (20 μM; C6I; 1 hour preincubation). The protein levels of STAT1, acetylated histone H3 and tubulin as loading control were determined by Western Blot analyses.C) Schematic representation of STAT1 protein structure highlighting its five functional domains and their amino acid boundaries. STAT1 also contains five putative caspase cleavage sites containing the DXXD, VXXD, or XXXD amino acid motifs. Numbers above the caspase cleavage sites correspond to their amino acid residues.D) *In vitro* cleavage assay of heterologously expressed His-STAT1 domains. STAT1 domains were assayed with caspase-3 or 6 as described in 4A. Western blot analysis was performed using anti-Penta-His antibody.

It is known that the immunodepletion of caspase-6 from apoptotic lysates cannot prevent the cleavage of STAT1 [27]. However, it has not been addressed whether caspase-6 may directly attack STAT1. To determine more thoroughly which caspase(s) can cleave STAT1 directly, we utilized GST-STAT1 as a substrate for the recombinant caspases 1, -3, -4, -6, -7, -8, and -9. Of the tested caspases, only caspase-3 and -6 cleave STAT1 at 1 U per reaction, yielding truncated fragments (Figure 4A; fragments marked with arrows), and this cleavage was inhibited by the pan-caspase inhibitor Z-VAD-FMK as well as by heat shock inactivation of the enzymes (Supplemental Figure 4.1).

To clarify whether STAT1 is also cleaved *in vivo* by caspase-3 and -6*,* we preincubated NB4 cells with cell permeable inhibitors for caspase-3 (zDEVD-FMK) or caspase-6 (zVEID-FMK), and treated the cells with butyrate (Figure 4B). Each of these caspase inhibitors prevented STAT1 cleavage, suggesting a critical role of caspase-3 and caspase-6 for the cleavage of STAT1.

Western blot analysis of cell extracts from NB4 cells revealed that the decrease in total STAT1 protein levels ties in with the appearance of one cleavage fragment of STAT1 (Figure 3E). However, recombinant STAT1 became cleaved into more fragments by caspases and these fragments could not be identified by antibodies (data not shown). Therefore, we concluded that multiple STAT1 fragments were generated as a consequence of caspase activation. Examination of the amino acid sequence of STAT1 with the GraBCas software and the CASVM server [46, 47] showed that STAT1 contains several potential cleavage sites being characterized by “DXXD,” “VXXD,” or “XXXD” sequence motifs; at amino acids 142, 167, 170, 694 with high probability (Figure 4C, supplement 3.1, supplement 3.2).

Therefore, we generated single mutants of the potential cleavage sites within STAT1 and performed an *in vitro* cleavage assay with caspase-3 and caspase-6. None of these STAT1 mutants was resistant to cleavage by these caspases (supplemental Figure 4.2), confirming the above mentioned *in silico* data illustrating that STAT1 contains more than one caspase cleavage site.

To verify the presence of multiple caspase cleavage sites in STAT1, we expressed and purified His-tagged STAT1 domains and repeated the *in vitro* caspase cleavage assay with recombinant caspase-3 and -6. The results revealed that STAT1 has caspase cleavage sites in the coiled-coiled domain, the DNA-binding domain as well as in the transactivation domain, and that both caspases are able to cleave STAT1 under these conditions (Figure 4D). These findings verify the above predicted absence of cleavage sites in its N-terminal domain and in the SH2 domain. Moreover, the data support the notion that STAT1 contains several caspase cleavage sites. Mass spectroscopy studies as well as Edman sequencing were performed with selected bands resulting from cleavage assays with GST tagged full length STAT1. These experiments verified that STAT1 is processed at multiple sites by caspase-3 and caspase-6 (supplemental Figure 4.3 4.4 and 4.5). Thus, STAT1 is a *bona fide* target of these enzymes.

### Butyrate leads to STAT1 degradation in leukemia cells but not in primary and differentiated cells

**Figure 5:**
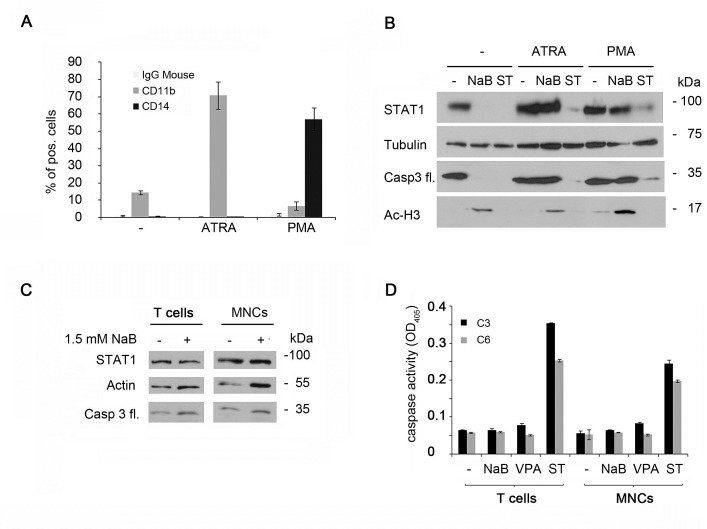
STAT1 degradation occurs exclusively in leukemia cells A) NB4 cells were stimulated with either 1 μM ATRA, 1 ng/ml PMA, or were left untreated (-) for 24 hours. Activity of these drugs was tested by flow cytometry with antibodies directed against the cell surface proteins CD11b and CD14 (means ± SE, n = 5). Mouse IgG served as control.B) Differentiation of NB4 cells stops the caspase-mediated STAT1 reduction. NB4 cells were preincubated with either 1 μM ATRA or 1 ng/ml PMA, or were left untreated (-) for 24 hours. Cells were then stimulated with 1.5 mM NaB, 200 nM staurosporine, or remained untreated (-). Levels of endogenous STAT1, tubulin, caspase-3 and acetylated histone H3 were monitored by immunoblot.C) STAT1 is not cleaved in primary cells. CD4^+^ T cells as well as MNCs from healthy donors were treated with 1.5 mM NaB or 200 nM staurosporine for 24 hours. Cell extracts were analyzed by Western blot analyses. D) Primary cells (5C) were stimulated with NaB (1.5 mM), VPA (1.5 mM) or staurosporine (200 nM) for 24 hours. Cells were lysated and the cell lysates were assayed for caspase-3 and caspase-6 activity with substrates containing classical caspase cleavage sites: DEVD-pNA (caspase-3) or VEID-pNA (caspase-6).

NB4 cells are promyelocytes that retain the bilineage potential. They are capable of maturation along the monocyte/macrophage lineage after phorbol myristate acetate (PMA) treatment or along the granulocytic maturation after induction with all-trans retinoic acid (ATRA) [48]. NB4 cell differentiation can be monitored by measuring the expression of the markers CD11b and CD14 on the cell surface. High levels of CD11b can be found after differentiation induced by ATRA, CD14 is increased upon differentiation by PMA (Figure 5A). NB4 cells were preincubated for 24 hours with ATRA or PMA and subsequently stimulated for 24 hours with butyrate or staurosporine, an apoptosis inducing agent [49, 50]. ATRA as well as PMA abolished the degradation of STAT1 by butyrate but not by staurosporine, indicating that differentiation specifically desensitizes cells against butyrate. Furthermore, caspase-3 cleavage was not detectable in NB4 cells treated with ATRA or PMA plus butyrate. In contrast, we could not identify any difference for staurosporine-induced STAT1 cleavage in undifferentiated and differentiated cells. In conclusion, we reason that undifferentiated cells have a higher sensitivity to the HDACi butyrate, which leads to a strong activation of caspases subsequently degrading STAT1 (Figure 5B).

So far, our data demonstrate that caspase-mediated STAT1 cleavage exclusively occurs in leukemia cell lines. Next, we tested if this effect could be observed in primary material. We assessed the HDACi sensitivity of human CD4-postive T cells and of a fraction of mono nuclear cells (MNCs). Surprisingly, in these cells there was no STAT1 reduction with 1.5 mM butyrate applied for 24 hours (Figure 5C). Even higher concentrations had no influence (supplemental Figure 5.1). To investigate this unexpected finding, we checked for caspase-3 cleavage in primary T cells and MNCs. Indeed, HDACi did not lead to caspase-3 activation. To assess the possibility that these agents may not be able to affect caspase-3 in primary cells, we treated them with staurosporine and measured caspase-3 activities in whole cell extracts. Staurosporine but not HDACi, like butyrate or VPA, activate caspase-3 and caspase-6 in T cells and MNCs (Figure 5D). Therefore, we conclude that primary cells are resistant to the induction of apoptosis by HDACi.

## DISCUSSION

STAT1 is overexpressed or constitutively activated in some types of leukemia and this can correlate with a bad prognosis for the patients [51, 52]. Therefore, there may be situations in which a more long term or permanent loss of STAT1 is beneficial. Previously, it was shown that apoptosis induced by exposure to etoposide resulted in a C-terminally truncated form of STAT1 [26]. Caspases were found to mediate this process in HeLa cells. Here, we report the cleavage of STAT1 by caspases in NB4 cells exposed to HDACi (Figures 1 and 2). We performed experiments with the HDACi butyrate, LBH-589 and MS-275, which have been described to induce apoptosis via the extrinsic as well as the intrinsic pathway (data not shown) [28, 53-55]. Depending on the nature of the apoptosis-inducing agent, different caspases are activated and this possibly results in diverse STAT1 cleavage patterns. Our data show that STAT1 is cleaved by the executioner caspases 3 and 6, generating multiple STAT1 fragments in NB4 cells exposed to butyrate (Figure 4A). This cleavage process can be prevented by blocking the enzymatic activities of caspase-3 and caspase-6 (Figure 4B). Whereas in some solid cancer cell lines STAT1 protein expression is induced after HDACi treatment, STAT1 is known to be acetylated and inactivated by HDACi [30]. Since leukemia cells are very sensitive to HDACi, in contrast to many cells derived from solid tumors, the cell type and perhaps the cellular context may determine the activation of distinct caspases and the impact on STAT1 signaling.

STAT1 and STAT3 share high similarity [30]. STAT3 is cleaved by caspases in prostate cancer cells generating multiple STAT3 protein fragments [56]. These STAT3 fragments retain transcriptional activity. We could show in *in vitro* cleavage assays that STAT1 is cleaved at several sites (Figure 4D and Supplemental Figure 4.3). The generated specific STAT1 cleavage products may contribute to STAT1 dependent gene expression in cells. This mechanism could represent a novel mechanism that links the activity of caspases to non-apoptotic cell functions. Further studies are required to find out whether caspase-generated STAT1 fragments play a role in gene transcription similar to those of STAT3

Several non-apoptotic functions of caspases have been identified in the last years, which indicate that caspases play an important role in cell differentiation and proliferation. Recent studies show that activation of caspases may selectively inactivate transcription factors, regulatory proteins and enzymes. For example, caspase-6 is able to cleave 5-lipoxygenase in B lymphocytic cells under non-apoptotic conditions [57]. Furthermore, caspase-3 is activated in terminally differentiated rodent lens epithelial cells and inhibition of caspase activity or overexpression of anti-apoptotic factors results in an abnormal development of cells [58]. The list of substrates cleaved by caspases under apoptotic and non-apoptotic conditions is still growing.

Both, the pan-caspase inhibitor Z-VAD-FMK as well as the caspase-3 inhibitor Z-DEVD-FMK allow the accumulation of STAT1 in NB4 cells. These data show for the first time that caspases control the stability of STAT1 in resting hematopoietic cells. We provide evidence suggesting that STAT1 is cleaved by caspases under apoptotic as well as under non-apoptotic conditions.

IFNs are commonly used as a therapy for cancer [59-61]. Consequently, STAT1 has been categorized as a pro-apoptotic factor within the IFNα/γ-dependent signaling cascades [62, 63]. Accordingly, studies with STAT1^−/−^ mice show that the loss of STAT1 increases the incidence of breast tumors [60]. Nevertheless, recent studies illustrate an ambivalent character of STAT1 [6, 64] and STAT1 can even accelerate the development of hematopoietic tumors independent of IFN signaling [8]. Interestingly, the upregulation of MHC class I molecules which is promoted by STAT1, represents a general mechanism how leukemic cells can escape tumor surveillance by natural killer cells in mice [6]. We consistently found that treating NB4 cells with butyrate causes a significant downregulation of the mRNA levels of *HLA-A* and *HLA-B (*Supplemental Figure 5.2*).* Truly, the cellular context may determine whether STAT1 acts as tumor promoter or as a tumor suppressor. The reduced levels of STAT1 caused by specific caspase cleavage may have an impact on the immune response and this could also affect cancer development. Our observations demonstrate that STAT1 is targeted by caspases upon HDACi treatment of leukemic cells. HDACi exposure of primary blood cells fails to induce caspase activation and STAT1 reduction (Figure 5C). These data may help to explain the beneficial effects of HDACi on tumor cells while sparing non-transformed cells [28].

In conclusion, HDACi induce the proteolytic processing of STAT1 by caspase-3 and caspase-6 in leukemic cells. This process is associated with the formation of several STAT1 cleavage fragments and it has implications on STAT1 target gene expression. The caspase-mediated processing of STAT1 under apoptotic and non-apoptotic conditions may represent an additional mechanism to modulate STAT1 signaling.

## METHODS

### Drugs and chemicals

Sigma-Aldrich: Butyrate, valproic acid, cycloheximide, staurosporine; Axxora: Caspase-1, -4 und -9, Z-VAD-FMK, Ac-DEVD-pNA, Ac-VEID-pna and Z-LLL-al (MG132); MERCK: Z-DEVD-FMK; Santa Cruz Biotechnology: Ac-VEID-FMK; Novartis: LBH589.

### Cell lines

NB4, THP-1, SD1, Nalm-6, Kasumi-1, BV173, Ramos, HeLa and primary cells were grown in RPMI supplemented with 10% FCS, 1% penicillin/streptomycin. SK-Mel-37, NW-Mel-1539, HEK-293T, HeLa, 2fTGH, CaCo2, HCT-116, and PC3 cells were maintained in DMEM containing the same additives and at 37°C in a 5% CO_2_ atmosphere.

### Plasmids

The following plasmids were used: vectors for active caspase-3, 6 and 8 were a gift from K. Lauber (Tübingen, Germany), caspase-3 subunits (p17-p12)_2_ were provided by B. Dälken and W. Wels (Frankfurt, Germany). GST-STAT1, a gift from I Behrmann (Luxemburg), was mutagenized using the Quick change site-directed mutagenesis kit (Stratagene). STAT1–domains were constructed the following way: pGEX-STAT1 was used as template and the fragments were cloned into pET19 with the CPEC–method [65]. All constructs were verified by DNA sequencing. Recombinant proteins were expressed in *E. coli BL21 (DE3)*. Primers are listed in Table.1 in the Supplemental Material section.

### Cell lysis and immunoblot

Cell lysis and immunoblot were performed as described in [25].

### Cleavage assay

Caspase-3 activity was measured as described [66]. GST-STAT1 was added to recombinant caspase-3 subunits or to caspase-6 subunits and incubated for 3-6 hours at 37°C.

### Antibodies

Antibodies used for immunoblot were purchased from Santa Cruz Biotechnology: Bcl-XL, #sc-634, p53 #sc-81168, STAT1 p84/p91 (E-23), #sc-346, STAT1α p91 (C-111), sc-417, STAT2 (C-20), #sc-476, STAT3 (C-20), #sc-482, Hsp90, #sc-13119, Caspase-3, #sc-7272, Caspase-6, #sc-1230 ; Sigma-Aldrich: Actin, A2066, Tubulin, #T5168; ‘9664; Upstate:H3-Ac, 06-599; BD Biosciences: PARP1, #556362 BD: FITC-AnnexinV,51-65874X; Qiagen: Penta-His Antibody 34660, EuroBioScience: PE-CD14, H12414P; Biozol: PE-CD11b, R0841; GeneTex: STAT1(internal) GTX113010 and STAT1 (SH2) GTX113011.

### Flow cytometry analyses, proliferation- and apoptosis assays

Information on these techniques can be found in [67] and [36].

## SUPPLEMENTARY FIGURES, TABLES AND METHODS


