# Supplementary material for: Caspase-3 and Caspase-6 cleave STAT1 in leukemic cells
Source: Oncotarget. 2014 Apr 18;5(8):2305–17. doi: 10.18632/oncotarget.1911 (PMC4039164; doi:10.18632/oncotarget.1911)
Supplement: Supplementary file 1 [file oncotarget-05-2305-s001.pdf]

Caspase-3 and Caspase-6 cleave STAT1 in leukemic cells

Material and Method for Supplementary Data

Table 1: Primers

Name	Sequence	Method
GAPDH	TGCACCACCAACTGCTTAGC, GGCATGGACTGTGGTCATGAG	qRT PCR
STAT1	CCGTTTTCATGACCTCCTGT, TGAATATTCCCCGACTGAGC	qRT PCR
STAT1_D143A	GACAAACAGAAAGAGCTTGCCAGTAAAGTCAGAAATGTG, CACATTTCTGACTTTACTGGCAAGCTCTTTCTGTTTGTC	Point mutants
STAT1_ D168A	GAGCCTGGAAGATTTACAAGCTGAATATGACTTCAAATGC,GCATTTGAAGTCATATTCAGCTTGTAAATCTTCCAGGCTC;	Point mutants
STAT1_ D171A	GATTTACAAGATGAATATGcCTTCAAATGCAAAACCTTG, CAAGGTTTTGCATTTGAAGGCATATTCATCTTGTAAATC;	Point mutants
STAT_ D694A	GCACCAGAGCCAATGGAACTTGCTGGCCCTAAAGGAACTGGATA, ATATCCAGTTCCTTTAGGGCCAGCAAGTTCCATTGGCTCTGGTGC	Point mutants
STAT1_D1	AGGGCCATATGGACCCATGTCTCAGTGGTACGAA, CCTCGAGGTCGACCCCCACTGTGCTCTGAATATT	STAT1 domain(1-134)
STAT1_D2	AGGGCCATATGGACCCATGTTAGACAACCAGAAA, CCTCGAGGTCGACCCCAAACGAGCTCTGAATGAG	STAT1 domain (135-317)
STAT1_D3	CCTCGAGGTCGACCCCCACTGTGCTCTGAATATT, AGGGCCATATGGACCCGTGGTGGAAAGACAGCCC	STAT1 domain (318-576)
STAT1_D4	CCTCGAGGTCGACCCCCCCATCATTCCAGAGAGG, AGGGCCATATGGACCCTGCATCATGGGCTTCATC	STAT1 domain (577-683)
STAT1_D5	CCTCGAGGTCGACCCCCCTGGAGTAATACTTTCC, AGGGCCATATGGACCCCCAAAGGAAGCACCAGAG.	STAT1 domain (684-749)

Details on primers for HLA-A and -B can be found in ref. [68].

Quantitative real- time PCR

mRNA was isolated and cDNA was synthesized as explained [39]. Primers are listed in Table 1. Data obtained from qRT-PCR were verified with the geNorm program.

Mass spectrometry

Protein bands were prepared for MS-analysis by proteolytic digestion with trypsin or V8-protease (GluC) according to Shevchenko et al. [69]. A LC-MS –system consisting of an Ettan-MDLC™-chromatography system (GE Healthcare, Munich, FRG) coupled to an ESI-ion trap masspectrometer (type LTQ, Thermo Electron, USA). Data analysis to identify STAT1-related tryptic- and GluC-specific peptides was performed with the Thermo Proteome Discoverer 1.0™ software ((Thermo Electron Corporation, Erlangen, Germany) the human.fasta protein database. For N-terminal sequencing of the processed STAT1 products the Procise 494A protein sequencer (Applied Biosystems, Foster City, CA, USA) was used.

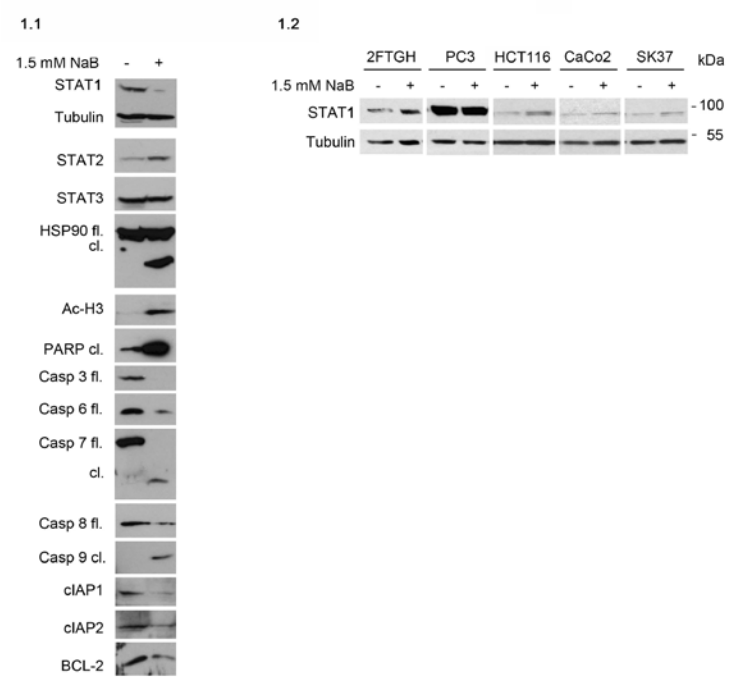

Supplemental Figure 1: 1.1 NB4 cells were treated with 1.5 mM NaB for 24 hours. Effects of apoptosis induction were studied. Indicated proteins were analysed by Western Blot

1.2 HDACi do not induce an apoptosis-dependent STAT1 reduction in cells derived from solid tumors (2FTGH, PC3, HCT116, CaCo2 and SK37). Actin were detected by Western blot analysis.

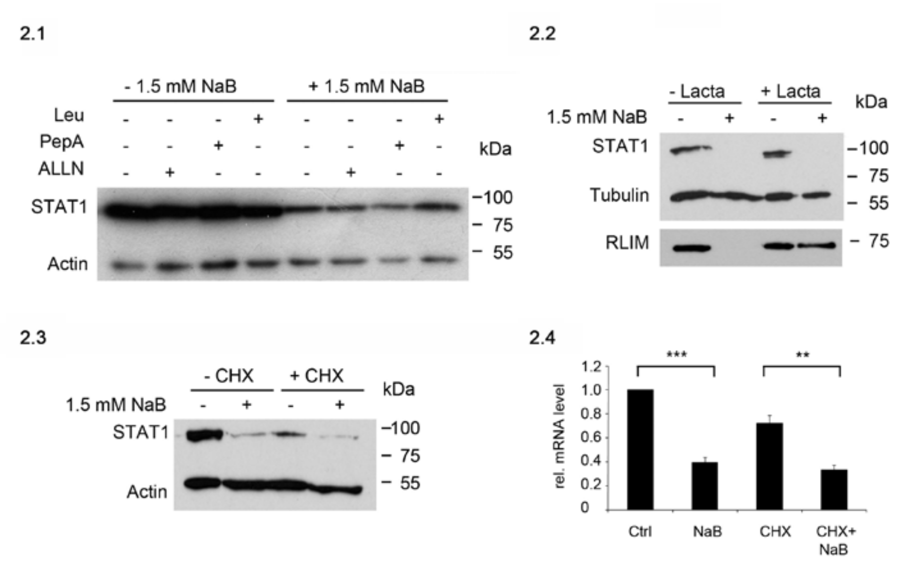

Supplemental Figure 2: 2.1 Proteases are not involved in the degradation of STAT1. NB4 cells were stimulated with 1.5 mM NaB in the absence or presence of the serine/threonine protease inhibitor leupeptin (10 µM), the aspartyl protease inhibitor pepstatin A (20 µM) and the calpain I and II as well as cathepsin B and L inhibitor ALLN (5 µM) for 24 hours (all inhibitors were given as 1 hour preincubation).

2.2 STAT1 is not degraded by the proteasome. NB4 cells were stimulated with 1.5 mM NaB in the absence or presence of the proteasome inhibitor lactacystin (5 µM, 4 h preincubation) for 24 hours. RLIM served as control for lactacystin.

2.3 STAT1 degradation does not require protein *de novo* synthesis. NB4 cells were stimulated with 1.5 mM NaB in the absence or presence of the translation inhibitor cycloheximide (10 µg/ml, 8 hours preincubation) for 24 hours. Protein levels were detected by immunoblot analyses as indicated.

2.4 Cycloheximide does not significantly affect mRNA levels of STAT1. NaB alone or combination of NaB and CHX resulted in reduced mRNA levels. (measns +/- SE; n=3, **p <0.01).

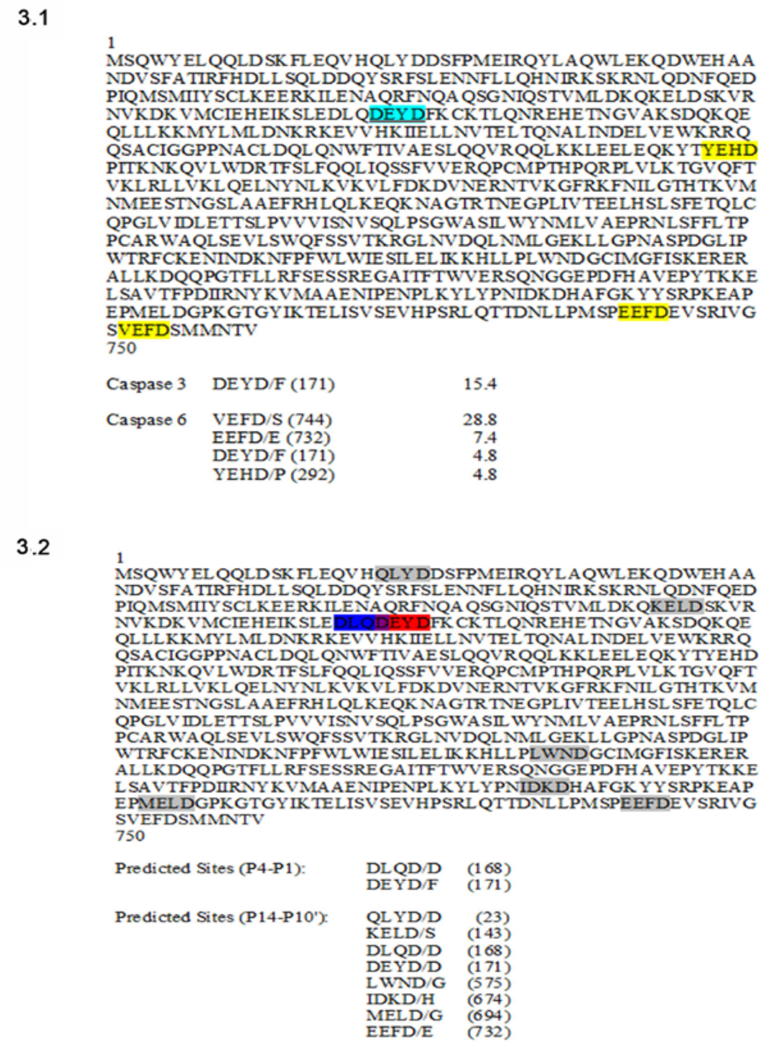

Supplemental Figure 3: 3.1 Examination of STAT1 protein sequence with the GraBCas software. Caspase cleavage site for caspase-3 is highlighted in blue. Caspase cleavage sites for caspase-6 are highlighted in yellow. Numbers in brackets show the position of the aspartate (P1). Numbers which are written after the cleavage sites show probability of these sequences. CC= coil-coiled domain; TAD= transcription activation domain

3.2 Examination of STAT1 protein sequence with CASVM server.

Caspase cleavage sites for caspase-3 are highlighted in blue and red. Numbers in brackets show the position of the aspartate (P1). Some non-canonical potential cleavage sites are highlighted in grey (MELD is proven). NTD= N-terminal domain; CC= coil-coiled domain; LD= linker domain; SH2= scr homology domain; TAD= transcription activation domain

4.1

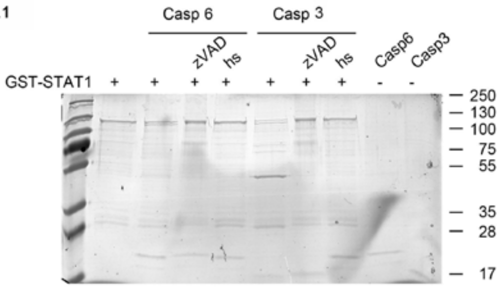
Casp 6
Casp 3
zVAD
hs
zVAD
hs
Casp6
Casp3
GST-STAT1 + + + + + + + - -
250
130
100
75
55
35
28
17

4.3

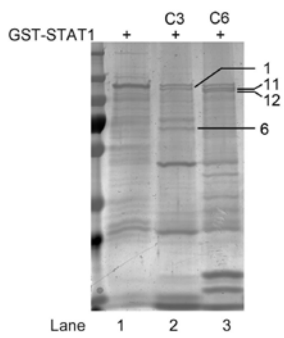
C3
C6
GST-STAT1 + + +
1
11
12
6
Lane 1 2 3

4.2

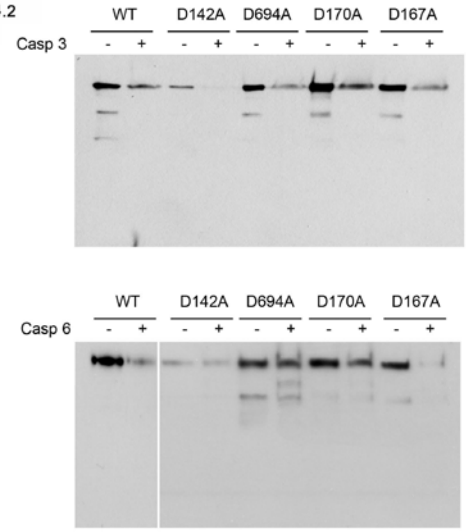
WT
D142A
D694A
D170A
D167A
Casp 3 - + - + - + - + - +
WT
D142A
D694A
D170A
D167A
Casp 6 - + - + - + - + - +

4.4

GST + Linker:

MSPILGYWKIKGLVQPTRLLLEYLEEKYEEHLYERDEGDKWRNKKFELGLEFPNLPYYIDGD
VKLTQSMAIIRYIADKHNMLGGCPKERAEISMLEGAVLDIRYGVSRIAYSKDFETLKVDFLS
KLPEMLKMFEDRLCHKTYLNGDHVTHPDFMLYDALDVVLYMDPMCLDAFPKLVCFKKRIEAI
PQIDKYLKSSKYIAWPLQGWQATFGGGDHPPKSDLIEGRGIPGIPIEFR

STAT1:

MSQWYELQQLDSKFLEQVHQLYDDSFPMEIRQYLAQWLEKQDWEHAANDVSFATIRFHDLLS
QLDDQYSRFSLENNFLLQHNIRKSKRNLQDNFQEDPIQMSMIIYSCLKEERKILENAQRFNQ
AQSGNIQSTVMLDKQKELDSKVRNVKDKVMCIEHEIKSLEDLQDEYDFKCKTLQNREHETNG
VAKSDQKQEQLLLKKMYLMLDNKRKEVVHKIIELLNVTELTQNALINDELVEWKRRQQSACI
GGPPNACLDQLQNWFTIVAESLQQVRQQLKKLEELEQKYTYEHDPITKNKQVLWDRTFSLFQ
QLIQSSFVVERQPCMPTHPQRPLVLKTGVQFTVKLRLLVKLQELNYNLKVKVLFDKDVNERN
TVKGFRKFNILGTHTKVMNMEESTNGSLAAEFRHLQLKEQKNAGTRTNEGPLIVTEELHSLS
FETQLCQPGLVIDLETTSLPVVVISNVSQLPSGWASILWYNMLVAEPRNLSFFLTPPCARWA
QLSEVLSWQFSSVTKRGLNVDQLNMLGEKLLGPNASPDGLIPWTRFCKENINDKNFPFWLWI
ESILELIKKHLLPLWNDGCIMGFISKERERALLKDQQPGTFLLRFSESSREGAITFTWVERS
QNGGEPDFHAVEPYTKKELSAVTFPDIIRNYKVMAAENIPENPLKYLYPNIDKDHAFGKYYS
RPKEAPEPMELDGPKGTGYIKTELISVSEVHPSRLQTTDNLLPMSPEEFDEVSRIVGSVEFD
SMMNTV

Colour	Domain	Colour	Domain
	GST-Tag + FXa-Cleavage site + Linker		NTD
	CC		DBD
	LD		SH2
	PY		TAD

4.5

Band	Caspase	Potentiell caspase cleavage site	calculated mol. Gewicht in kDa	N-terminus
1	3	NTD, CC,TAD	112	MSP
6	3	NTD, CC,SH2	57-63	FKCKT
11	6	PY	103	MSP
12	6	PY	103	

NTD= N-terminal domain; CC= coiled-coil domain; DBD= DNA binding domain; LD= linker domain; SH2= src homology domain; PY= phosphorylation site; TAD= transcription activation domain

Supplemental Figure 4: 4.1 Cleavage of STAT1 can be inhibited by caspase inhibitors. GST-STAT1 was incubated either with caspase-3 or caspase-6 (1 unit). As control served pan-caspase inhibitor Z-VAD-FMK (zVAD) as well as heat shock (hs) inactivating of caspase activity. Extracts were analyzed by SDS-PAGE and stained with Coomassie solution.

4.2 Potential cleavage sites within the STAT1 protein were changed. Asparagine was mutated to Alanine. WT-GST-STAT1 as well as single mutants were assayed with 1 unit either caspase-3 or caspase-6 for 3 hours at 37° C. Western blot analysis was performed by the usage of a STAT1 antibody.

4.3 Proteolytic processing *in vitro* of GST-STAT1 by caspase-3 and caspase-6. The experiments were performed in three independent experiments. Lane 1: Untreated GST-STAT1. A single step batch

procedure with GSH-Sepharose was used to prepare the protein. Numerous protein bands below the mature GST-STAT1 protein with a molecular weight of approximately 120 kDa are seen. Selected bands were checked for protein identity by mass spectrometry. Obviously all of them represent GST-STAT1 degradation products that occurred during protein expression and/or purification. In contrast, no *E.coli* proteins were among them. Lanes 2 and 3: GST-STAT1 treated with caspase-3 and -6, respectively. Treatments were performed in caspase cleavage buffer for 6 h at 37° C. This point in time was selected after evaluation of the kinetics of caspase processing (data not shown).

Mass spectrometry analyses were performed with selected bands, marked with arrows in **Supplemental Figure 4.3**. Comparison of the protein pattern suggests that only a limited number of protein bands in lanes 2 and 3 really refer to GST-STAT 1 protein processing products induced by treatments with caspase-3 and caspase-6. To determine the particular cleavage areas of caspases we assessed the N-terminal and C-terminal endpoints of the polypeptide products, i.e. the maximum span of the polypeptide chain. To this end we attempted to localize the tryptic- and V8-protease proteolytic peptides prepared from the protein bands within the GST-STAT1 sequence by mass spectrometry. As an alternative approach the N-termini were determined by N-terminal sequencing by Edman degradation after blotting the protein gels on PVDF membranes.

Unfortunately, for some selected bands no interpretable MS- and/or N-terminal sequencing results could be obtained. Therefore, we are able to present results for a restricted number of bands indicated in **Supplemental Figure 4.3** with numbers 1 and 6 (caspase-3 treatment) and with numbers 11, 12 (caspase-6 treatment). The drawing below demonstrates the principle analytical approach with band 1 as an example:

The partial amino acid sequence of the GST-tag including a linker sequence and the functional domains of STAT1 are highlighted by different colors as indicated in the legend. The N-terminus detected by sequencing is shown in bold blue letters; tryptic- and V8-peptides are shown in bold red and yellow letters, respectively S**upplemental Figure 4.4**.

By N terminal sequencing the N-terminus of the GST-STAT1 was detected (**MSPIL**). The tryptic-peptides detected by MS were found to be distributed nearly over the whole (remaining) protein sequence, while the V8-peptide **VHPSRLQTTDNLLPMSPE** was the most C-terminally located peptide. The conclusion is: Caspase-3-processind occurred in the polypeptide stretch that follows the V8-peptide, i.e. the caspase-3-cleavage site (area) is located in the transcriptional activation domain. The calculated molecular weight of the protein fragment is about 112 kDa. Using this approach the caspase-3 and caspase-6 cleavages sites were located (narrowed, described) for all selected protein bands. The results are summarized in the below-mentioned table (**Supplemental Figure 4.5**).

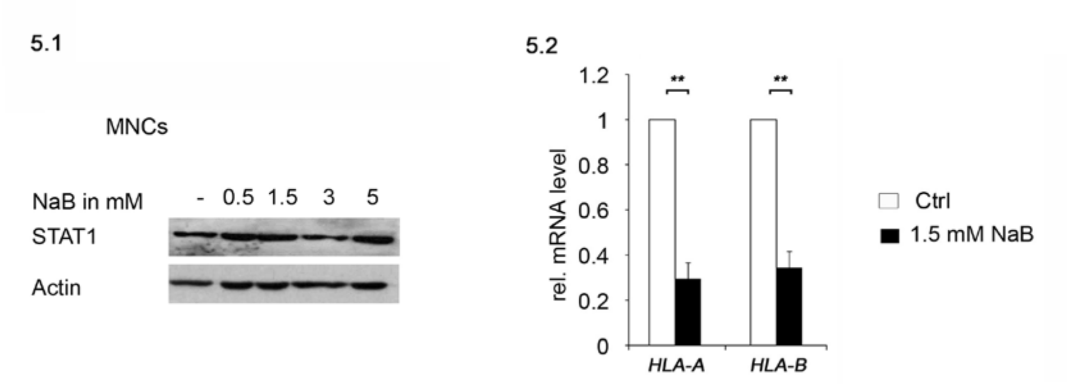

Supplemental Figure 5: 5.1 MNCs which were stimulated with NaB do not degrade STAT1. MNCs were treated with 0-5 mM NaB for 24 hours. Expression levels of STAT1 and Actin levels were analyzed by Western Blot.

5.2 NB4 cells were treated with 1.5 mM Butyrate for 24 hours. Expression of HLA-A and HLA-B mRNA was analyzed by quantitative real-time PCR. Relative expression relates to corresponding values for samples from untreated cells, set as 1 (means ± SE; n = 3; **p < 0.01)
